# Healthcare resource utilization and costs in pediatric patients under 6 years old with atopic dermatitis in the United States: A retrospective, observational, cross-sectional study

**DOI:** 10.1016/j.jdin.2026.04.025

**Published:** 2026-06-19

**Authors:** Jonathan I. Silverberg, Bruno Martins, Mengtian Du, Min Yang, Kerry Noonan, Andrew Korotzer, Zhixiao Wang

**Affiliations:** a George Washington University School of Medicine and Health Sciences, Washington, District of Columbia; b Analysis Group, Boston, Massachusetts; c Sanofi, Cambridge, Massachusetts; d Regeneron Pharmaceuticals Inc., Tarrytown, New York

**Keywords:** atopic dermatitis, disease burden, epidemiology, healthcare costs, healthcare resource utilization, pediatric patients

## Abstract

**Background:**

Atopic dermatitis (AD) (also known as eczema) is a long-term skin disease affecting 13% of children in the United States. These children often have other inflammatory conditions, including asthma, hay fever (allergic rhinitis), and food allergies. Currently, little is known about the use of healthcare resources (hospital visits and admissions and emergency room/urgent care visits) and the associated healthcare costs in children with AD. In this study, we looked at the treatment received for AD, use of healthcare, and associated costs in patients with AD in the United States aged between 6 months and 6 years who were treated for their AD. Patients were split into 3 groups based on received treatments: low-potency topical treatment (LTT) used to treat mild AD, medium-to-high-potency topical treatment (MHTT) for moderate AD, and systemic treatments (ST) for severe AD. Of 23,098 patients studied, 5,452 received LTT, 6,778 received MHTT, and 10,868 received ST. The use of healthcare was similar in children treated with LTT and MHTT, but higher in children treated with ST, including hospital admissions and emergency room/urgent care visits. Healthcare costs per patient were higher in AD patients treated with MHTT and ST, compared to LTT, primarily due to medical expenses. Children had a high number of AD-related inflammatory conditions, with highest burden in the ST group The study showed that patients treated with ST had higher healthcare utilization and costs, suggesting a higher disease burden.

**Objective:**

To assess healthcare resource utilization and healthcare costs in this patient group.

**Methods:**

This retrospective, 1-year, cross-sectional, observational study (July 2016-December 2019) of US patients used data from the IQVIA PharMetrics Plus Database. Patients were assigned to cohorts based on treatments received: LTTs, MHTTs, and STs.

**Results:**

Overall, 23,098 patients were included (LTT: 5452; MHTT: 6778; ST: 10,868). All-cause health care resource utilization was generally similar for the MHTT and LTT cohorts, but was higher in the ST cohort, including inpatient admissions (1.5% and 1.5% vs 6.5%) and emergency room/urgent care visits (22.0% and 22.0% vs 37.5%). The mean all-cause healthcare costs per patient were higher in the ST and MHTT cohorts vs LTT cohort ($9272.24 and $5671.60 vs $5249.69), with the majority being medical costs ($8135.93 and $4669.39 vs $4221.56).

**Limitations:**

Possibility of selection bias and misclassification if treatments were used for conditions other than atopic dermatitis; exclusion of patients using over-the-counter treatments; neglecting out-of-pocket costs.

**Conclusion:**

Patients receiving ST had higher healthcare resource utilization and costs than patients receiving LTT, with medical costs being the main driver.

**Capsule Summary**

• Real-world evidence of healthcare resource utilization and treatment patterns in pediatric patients under 6 years old with atopic dermatitis is limited.
• In this study, patients receiving systemic treatments for severe atopic dermatitis had higher healthcare resource utilization and costs; a substantial proportion of pediatric patients were receiving systemic corticosteroids.

## Introduction

Atopic dermatitis (AD), a chronic skin disorder affecting up to 13% of the US population aged <18 years, typically manifests as red, itchy, swollen, cracked skin, and often compromises patients’ health-related quality of life.^1^ The estimated prevalence of having had AD among children aged <5 years is between 15% and 38%,^2^ with an estimated 12-month prevalence of 10.2% among children aged <6 years and an estimated 46.5% mild, 42.6% moderate, and 10.8% severe cases among children aged 6 months to <6 years who have been diagnosed with AD in the United States.^3^

For infants and children aged ≥3 months with AD of any severity, the 2023 guidelines issued by the American Academy/American College of Allergy, Asthma & Immunology Joint Task Force recommend initial treatment with prescription moisturizers.^4^ For patients with mild-to-moderate AD refractory to moisturizers, topical phosphodiesterase-4 inhibitors (such as crisaborole) are recommended; for those with mild-to-severe AD, topical corticosteroids (TCSs) or topical calcineurin inhibitors are recommended. Options are limited for infants and young children who are refractory to, intolerant of, or unable to use medium-to-high-potency topical treatments (MHTTs). The only systemic treatments (STs) currently recommended for use in children with moderate-to-severe AD (aged ≥6 months) are dupilumab in multiple countries and regions, including the United States and Europe,^4^, ^5^, ^6^, ^7^ and baricitinib (age ≥2 years), only approved in Europe,^8^ and the guidelines recommend against the use of systemic corticosteroids (SCSs) for all patients with AD.

There are currently no published studies on the burden of AD, in terms of healthcare resource utilization (HRU) and healthcare costs, in pediatric patients aged <6 years in a real-world setting.

This study assessed the patient characteristics, HRU, and healthcare costs in pediatric patients aged ≥6 months to <6 years with AD overall and stratified by age group (≥6 months to <2 years vs ≥2 to <6 years).

## Methods

### Study design

This retrospective, cross-sectional, observational study, between July 1, 2016, and December 31, 2019, was conducted prior to the approval of dupilumab for use in children. We confined the study to the pre-COVID-19 period to avoid potential confounding factors or bias on HRU patterns caused by the COVID-19 pandemic.

The index date for a study participant was defined as January 1 of the eligible calendar year for observation. If a participant had multiple eligible calendar years, a single year was randomly selected. The observation period for a study participant was defined as January 1 to December 31 of the eligible calendar year (12 months) in 2017, 2018, or 2019.

### Data source

Study data were obtained from the IQVIA PharMetrics Plus Database,^9^ a longitudinal health plan database of adjudicated medical and pharmacy claims in the United States that includes patient enrollment data for national and subnational health plans and self-insured employer groups. The database has included >200 million enrollees since 2006 and provides adequate representation of all areas across the United States. Available data include demographic characteristics, inpatient (IP) and outpatient (OP) diagnoses and procedures, OP prescriptions, and costs paid by health plans to providers.

### Patients

Patients aged ≥6 months to <6 years on the index date with at least 1 medical diagnosis claim for AD during the eligible calendar year and 6 months before the eligible calendar year were included to ensure that they had been diagnosed with AD during the entire observational period (Supplementary Fig 1, available via Mendeley at https://data.mendeley.com/datasets/6ntkw3zv3f/1). Patients needed to have continuous enrollment in the database for the calendar year and at least 6 months before the calendar year, and at least 1 claim during the calendar year, for any of the following treatments: TCS, topical calcineurin inhibitor, crisaborole, SCS, systemic immunosuppressants, dupilumab, intravenous immunoglobulin, or phototherapy. The observational period for each patient was 12 months (1 calendar year). Patients with AD who were not prescribed treatment were excluded.

Patients were categorized into 3 cohorts by treatments received during the calendar year based on criteria adapted from Paller et al^10^: low-potency topical treatments (LTTs), MHTTs, and ST.

The LTT cohort received LTTs, topical calcineurin inhibitors, or crisaborole; the MHTT cohort received MHTTs; and the ST cohort received STs: SCS, systemic immunosuppressants (azathioprine, cyclosporine A, interferon γ, methotrexate, mycophenolate mofetil), dupilumab (approved by the US Food and Drug Administration in June 2022 for patients with AD aged <6 years), intravenous immunoglobulin, or phototherapy.

Patients with treatments from multiple categories within the calendar year were categorized according to the highest potency of prescribed treatments (eg, a patient prescribed both crisaborole and SCS would be categorized into the ST cohort).

### Variables

Variables included patient characteristics (age, sex, geographic region, insurance type, comorbidity profile), HRU (data on IP admissions [any IP admission, number of IP admissions, length of stay], emergency room [ER] or urgent care [UC] visits [any ER/UC visit, number of ER/UC visits], and OP visits [any OP visit, number of OP visits]), and healthcare costs (total healthcare costs, pharmacy costs, medical costs [IP, ER/UC, OP costs]). These analyses do not include patient out-of-pocket costs, as these are not captured by the claims data. All costs are annualized per patient and were inflated to 2021 US dollars (the first year following the end of the data period, excluding 2020 to avoid any COVID effects).

### Statistical analyses

#### Descriptive analyses

In the descriptive analyses, variables were summarized descriptively in the observation period by treatment cohorts (LTT, MHTT, ST), including patient characteristics, HRU (all-cause and AD-related), and healthcare costs (all-cause and AD-related). Patient characteristics, HRU, and healthcare costs were also summarized descriptively for the following age subgroups: ≥6 months to <2 years and ≥2 to <6 years. Descriptive analyses were conducted by AD treatment cohort within each age subgroup.

Unadjusted statistical comparisons across AD treatment cohorts (using the LTT cohort as a reference) within each subgroup were conducted using Wilcoxon rank sum tests for continuous variables, and chi-square/Fisher’s exact tests for categorical variables. A *P* value < .05 was regarded as significant. Statistical analyses were performed using SAS Enterprise Guide version 7.1 (SAS Institute).

#### Adjusted analyses

Adjusted all-cause healthcare costs (total healthcare costs, medical costs, pharmacy costs) in the observational period were estimated for each AD treatment cohort. An inverse probability of treatment weighting approach was used. Each patient was weighted by the inverse of the adjusted probability of being in the assigned treatment cohort given the following covariates: age at index (calculated based on year of birth, because the IQVIA PharMetrics Plus Database does not include the day or month of birth; ≤1, 2, 3, 4, 5 years), sex (male, female), region (South, Midwest, Northeast, West, unknown), plan payer (commercial, other), plan type (preferred provider organization, other), number of comorbidities, and presence of food allergy (FA), allergic rhinitis (AR), asthma, or allergic urticaria.

## Results

Among 523,086 pediatric patients (aged ≥6 months to <6 years) with at least 1 diagnosis of AD between January 2017 and December 2019 included in the IQVIA PharMetrics Plus Database, 54,775 had continuous enrollment in the calendar year and at least 6 months before the calendar year, 31,677 (57.8%) did not report any prescribed treatment and were excluded from the analysis, and 23,098 received AD treatment and met the full study inclusion criteria (Supplementary Fig 2, available via Mendeley at https://data.mendeley.com/datasets/6ntkw3zv3f/1).

Of the included patients, 5452 were assigned to the LTT cohort (<2 years: 1672; ≥2 years: 3780), 6778 to MHTT (<2 years: 1462; ≥2 years: 5316), and 10,868 to ST (<2 years: 2483; ≥2 years: 8385) (Supplementary Tables I, *A* and *B*, available via Mendeley at https://data.mendeley.com/datasets/6ntkw3zv3f/1).

### Patient characteristics

**Fig 1:**
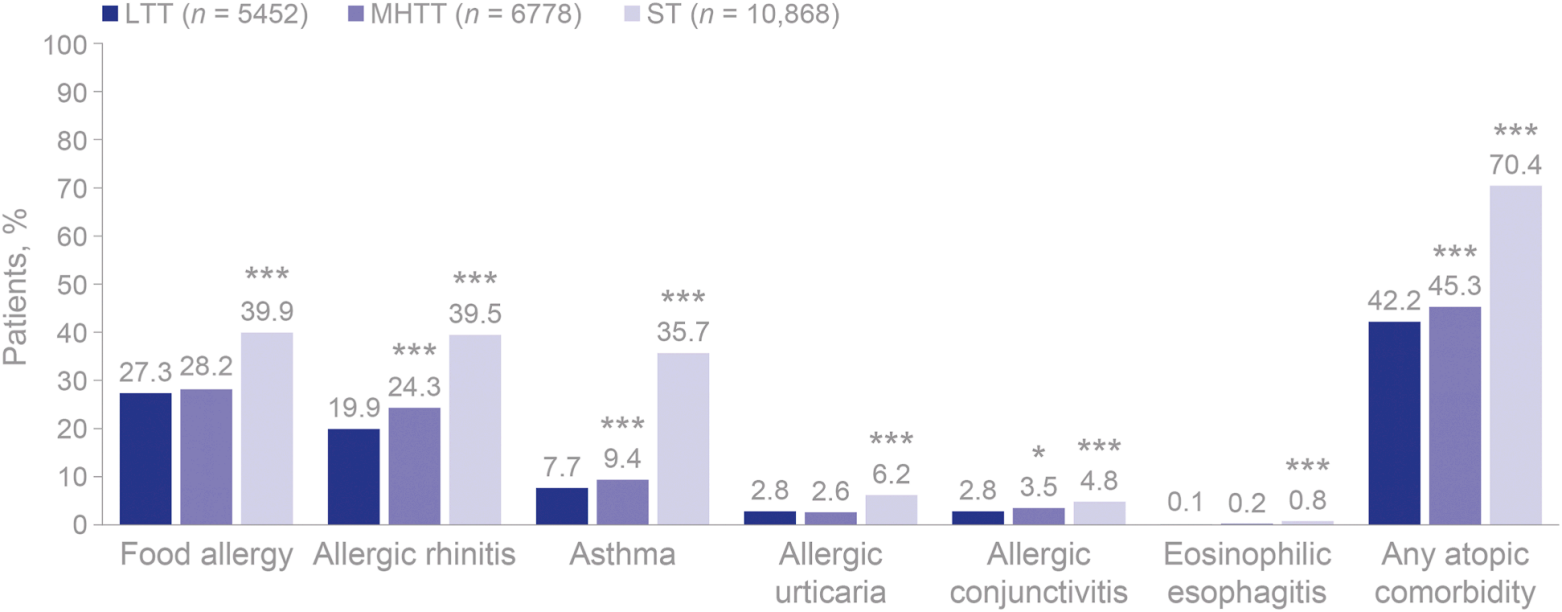
Proportion of pediatric patients with atopic dermatitis and atopic comorbidities by treatment cohort. Atopic comorbidities included allergic rhinitis, allergic conjunctivitis, food allergy, allergic urticaria, asthma, eosinophilic esophagitis, and nasal polyps. *LTT*, Low-potency topical treatment; *MHTT*, medium-to-high-potency topical treatment; *ST*, systemic treatment. ∗∗∗*P* < .001; ∗*P* < .05 vs LTT.

Characteristics of patients in the ST and MHTT cohorts were significantly different from those of patients in the LTT cohort (Table I). The mean (standard deviation [SD]) age of patients receiving ST or MHTT was significantly greater than that of patients receiving LTT (2.58 [1.30] and 2.66 [1.31] vs 2.35 [1.27], respectively, *P* < .001 MHTT/ST vs LTT). Patients receiving ST were less likely than those receiving MHTT and LTT to be female (35.1% vs 42.7% and 43.9%, respectively), and more likely to be enrolled in a preferred provider organization plan (81.8% vs 77.2% and 75.2%, respectively, *P* < .001 ST vs LTT). Patients receiving ST or MHTT were significantly more likely than those receiving LTT to have any atopic comorbidity, allergic conjunctivitis, asthma, or AR; patients receiving ST were more likely than those in other cohorts to have eosinophilic esophagitis, allergic urticaria, or FA (Fig 1).

**Table I:** Patient characteristics and comorbidities

	Treatment cohorts				
	LTT	MHTT	*P* value	ST	*P* value
	*n* = 5452	*n* = 6778		*n* = 10,868	
Demographic characteristics					
Age at index date, years, mean ± SD	2.35 ± 1.27	2.66 ± 1.31	**<.001**	2.58 ± 1.30	**<.001**
Age categories, *n* (%)			**<.001**		**<.001**
Less than 2 y old	1672 (30.7)	1462 (21.6)		2483 (22.8)	
2 y or older	3780 (69.3)	5316 (78.4)		8385 (77.2)	
Female, *n* (%)	2394 (43.9)	2896 (42.7)	.19	3819 (35.1)	**<.001**
Index year, *n* (%)			**<.001**		**<.001**
2017	1582 (29.0)	2199 (32.4)		3291 (30.3)	
2018	1877 (34.4)	2202 (32.5)		3418 (31.5)	
2019	1993 (36.6)	2377 (35.1)		4159 (38.3)	
Region, *n* (%)			**<.001**		**<.001**
South	1980 (36.3)	2964 (43.7)		5388 (49.6)	
Midwest	1372 (25.2)	1484 (21.9)		2562 (23.6)	
Northeast	1358 (24.9)	1434 (21.2)		1909 (17.6)	
West	739 (13.6)	896 (13.2)		1006 (9.3)	
Insurance plan characteristics					
Plan type,∗*n* (%)			**.04**		**<.001**
PPO	4098 (75.2)	5231 (77.2)		8890 (81.8)	
HMO	831 (15.2)	979 (14.4)		1120 (10.3)	
POS	393 (7.2)	414 (6.1)		613 (5.6)	
CDHP	114 (2.1)	139 (2.1)		206 (1.9)	
Indemnity	47 (0.9)	49 (0.7)		107 (1.0)	
Unknown	17 (0.3)	26 (0.4)		28 (0.3)	
Number of comorbidities, mean ± SD	0.78 ± 1.06	0.88 ± 1.13	**<.001**	1.82 ± 1.51	**<.001**
Number of comorbidities, *n* (%)			**<.001**		**<.001**
0	2941 (53.9)	3410 (50.3)		2751 (25.3)	
1	1410 (25.9)	1773 (26.2)		2308 (21.2)	
2	698 (12.8)	954 (14.1)		2300 (21.2)	
3	238 (4.4)	383 (5.7)		1815 (16.7)	
4+	165 (3.0)	258 (3.8)		1694 (15.6)	
Atopic comorbidities,† mean ± SD	2301 (42.2)	3072 (45.3)	**<.001**	7653 (70.4)	**<.001**

Bold *P* values indicate statistically significant differences vs LTT.

*CDHP*, Consumer-directed healthcare plan; *HMO*, health maintenance organization; *LTT*, low-potency topical treatment; *MHTT*, medium-to-high-potency topical treatment; *POS*, point of service; *PPO*, preferred provider organization; *SD*, standard deviation; *ST*, systemic treatment.

∗ Not mutually exclusive, due to patients having multiple plans in the year.

† Atopic comorbidities include allergic rhinitis, allergic conjunctivitis, food allergy, allergic urticaria, asthma, eosinophilic esophagitis, and nasal polyps.

In subgroup analyses, patients in the <2-year group had a similar number of any atopic comorbidity, FA, AR, asthma, allergic urticaria, allergic conjunctivitis, and eosinophilic esophagitis in the LTT and MHTT cohorts, but that number was significantly higher in the ST cohort (vs LTT) for all atopic comorbidities. In the ≥2-year group, patients receiving ST or MHTT were significantly more likely than those receiving LTT to have any atopic comorbidity, AR, or asthma; patients receiving ST also had more FA, allergic urticaria, allergic conjunctivitis, or eosinophilic esophagitis than those receiving LTT or MHTT (Supplementary Table II, available via Mendeley at https://data.mendeley.com/datasets/6ntkw3zv3f/1).

### Healthcare resource utilization

All-cause HRU was generally similar for patients in the MHTT and LTT cohorts, but significantly higher in the ST cohort, including for IP admission (1.5% and 1.5% vs 6.5%, respectively) and ER/UC visits (22.0% and 22.0% vs 37.5%, respectively, *P* < .001 ST vs LTT) (Table II).

**Table II:** All-cause HRU of pediatric patients with AD by treatment

	Treatment cohorts				
	LTT	MHTT	*P* value	ST	*P* value
	*n* = 5452	*n* = 6778		*n* = 10,868	
All-cause HRU					
IP					
Any IP admission, *n* (%)	81 (1.5)	103 (1.5)	.88	708 (6.5)	**<.001**
Number of IP admissions, mean ± SD	0.02 ± 0.18	0.02 ± 0.15	.44	0.08 ± 0.38	**<.001**
Length of stay, mean ± SD	0.04 ± 0.60	0.05 ± 0.65	.39	0.23 ± 2.43	**<.001**
ER or UC					
Any ER or UC visit, *n* (%)	1199 (22.0)	1489 (22.0)	.97	4079 (37.5)	**<.001**
Number of ER or UC visit, mean ± SD	0.35 ± 0.83	0.35 ± 0.83	.45	0.76 ± 1.36	**<.001**
OP					
Any OP visit, *n* (%)	5452 (100.0)	6777 (99.9)	1.00	10,868 (100.0)	–
Number of OP visit, mean ± SD	10.82 ± 14.02	10.75 ± 14.31	**<.01**	15.73 ± 17.46	**<.001**

Bold *P* values indicate statistically significant differences vs LTT.

*AD*, Atopic dermatitis; *ER*, emergency room; *HRU*, healthcare resource utilization; *IP*, inpatient; *LTT*, low-potency topical treatment; *MHTT*, medium-to-high-potency topical treatment; *OP*, outpatient; *SD*, standard deviation; *ST*, systemic treatment; *UC*, urgent care.

Although almost all included patients had OP visit(s) during the observation period, partially due to patients requiring an AD diagnosis during the calendar year, the mean (SD) number of OP visits was significantly higher in the ST versus LTT cohort (15.73 [17.46] vs 10.82 [14.02], *P* < .001), but lower in the MHTT cohort (10.75 [14.31], *P* < .01) (Table II).

In subgroup analyses, all-cause and AD-related HRU was mostly similar in the MHTT and LTT cohorts in both age subgroups (<2 years vs ≥2 years), but significantly higher in the ST cohort in both subgroups (Supplementary Tables III and IV, available via Mendeley at https://data.mendeley.com/datasets/6ntkw3zv3f/1).

All IP and ER/UC are also shown per 1000 patients (Supplementary Table V, available via Mendeley at https://data.mendeley.com/datasets/6ntkw3zv3f/1).

### Healthcare costs

**Fig 2:**
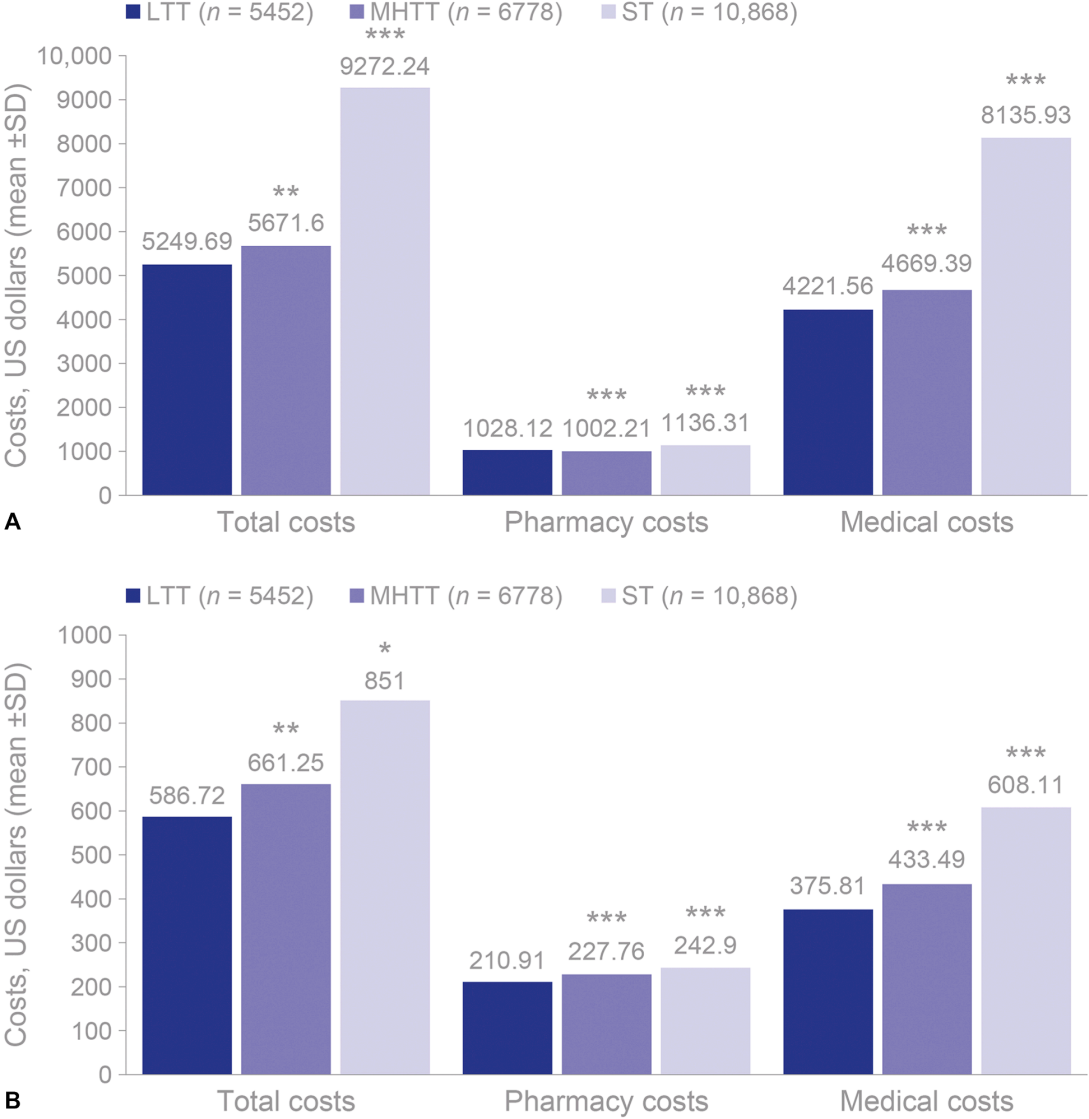
All-cause^a^**(A)** and AD-related **(B)** healthcare costs of pediatric patients with AD by treatment cohort.^b^*AD*, Atopic dermatitis; *LTT*, low-potency topical treatment; *MHTT*, medium-to-high-potency topical treatment; *SD*, standard deviation; *ST*, systemic treatment. ∗∗∗*P* < .001; ∗∗*P* < .01; ∗*P* < .05 vs LTT. ^a^Adjusted all-cause healthcare costs (total healthcare costs, medical costs, pharmacy costs) in the observational period were estimated for each AD treatment cohort. An inverse-probability-of-treatment-weighting approach was used. Each patient was weighted by the inverse of the adjusted probability of being in the assigned treatment cohort given the following covariates: age at index (calculated based on year of birth because the IQVIA PharMetrics Plus Database does not include the day and month of birth) (≤1, 2, 3, 4, 5 years), sex (male, female), region (South, Midwest, Northeast, West, unknown), plan payer (commercial, other), plan type (preferred provider organization, other), number of comorbidities, and presence of food allergy, allergic rhinitis, asthma, or allergic urticaria. ^b^Healthcare resource utilization and healthcare costs were measured during the 12 months after the index date (the index date is defined as the first calendar day of the year).

The mean all-cause healthcare costs per patient were significantly higher in the ST and MHTT cohorts versus the LTT cohort, including total costs ($9272.24 and $5671.60 vs $5249.69, respectively, *P* < .001 ST vs LTT, *P* < .01 MHTT vs LTT) and medical costs ($8135.93 and $4669.39 vs $4221.56, respectively, *P* < .001 ST/MHTT vs LTT) (Fig 2, *A*). The mean AD-related healthcare costs per patient were significantly higher in the ST and MHTT cohorts versus the LTT cohort, including total costs ($851.00 and $661.25 vs $586.72, respectively, *P* < .05 ST vs LTT, *P* < .01 MHTT vs LTT), pharmacy costs ($242.90 and $227.76 vs $210.91, *P* < .001 ST/MHTT vs LTT), and medical costs ($608.11 and $433.49 vs $375.81, *P* < .001 ST/MHTT vs LTT) (Fig 2, *B*).

**Fig 3:**
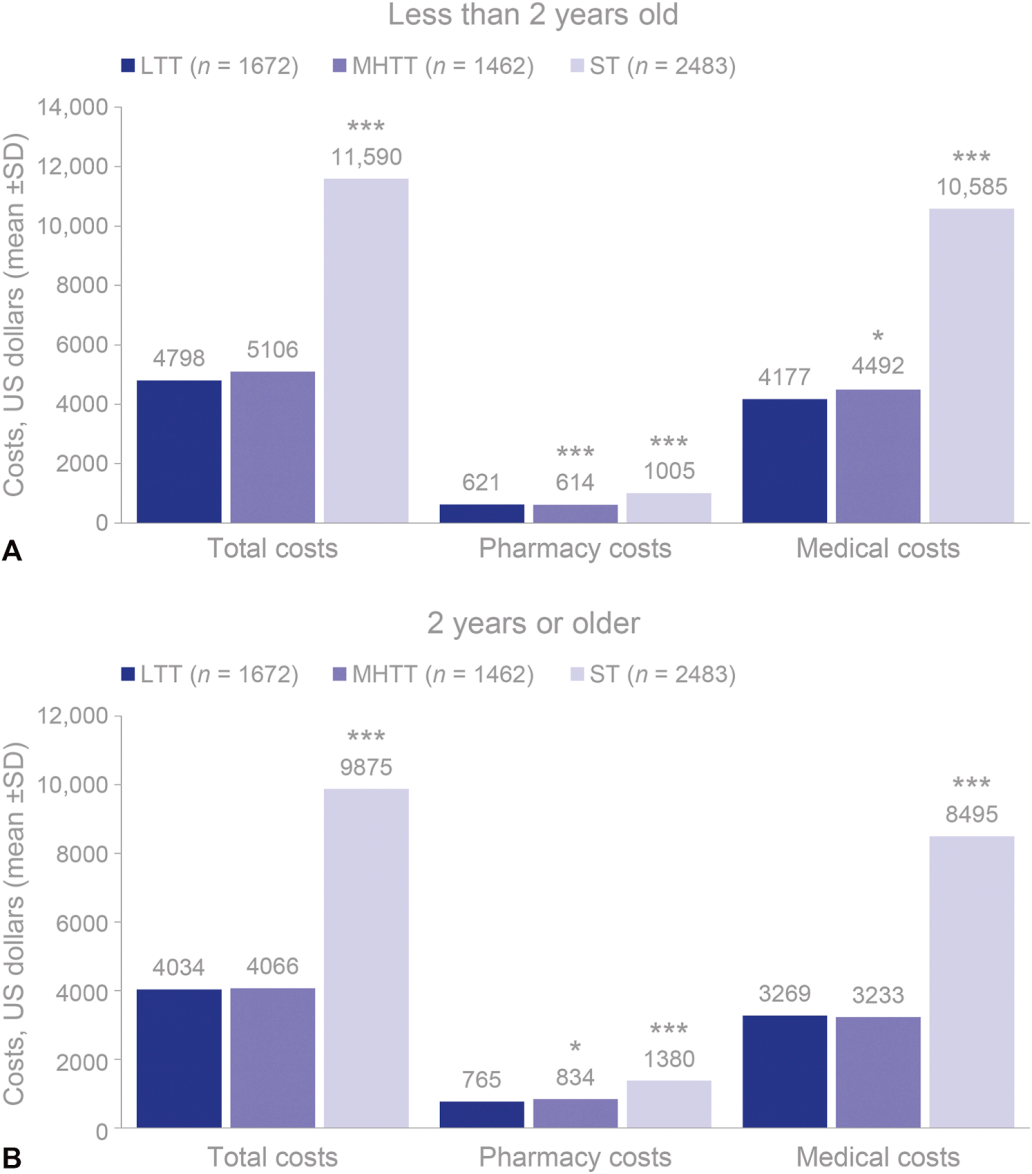
All-cause healthcare costs of pediatric patients with atopic dermatitis by age category. **A,** Patients less than 2 years old and **B**, Patients 2 years or older. ^a^ ∗∗∗*P* < .001; ∗*P* < .05 vs LTT. ^a^Healthcare resource utilization and healthcare costs were measured during the 12 months after the index date (the index date is defined as the first calendar day of the year). All presented costs are annualized per patient. *LTT*, Low-potency topical treatment; *MHTT*, medium-to-high-potency topical treatment; *SD*, standard deviation; *ST*, systemic treatment.

A similar pattern of all-cause healthcare costs was found in both age subgroups (<2 years vs ≥2 years), with considerably higher costs in the ST cohort versus the MHTT and LT cohorts (Fig 3).

In subgroup analyses of AD-related healthcare costs, overall AD-related healthcare medical costs were higher with severity cohort in both age subgroups (Supplementary Table VI, available via Mendeley at https://data.mendeley.com/datasets/6ntkw3zv3f/1). Furthermore, patients in the <2-year subgroup had higher total costs across all cohorts than patients in the older subgroup.

## Discussion

This study characterized pediatric patients aged ≥6 months to <6 years with AD in the United States and quantified their economic burden in terms of HRU and healthcare costs according to treatments received during the calendar year (LTT, MHTT, and ST). Patients in the ST cohort had a higher prevalence of comorbidities than patients in the LTT or MHTT cohorts, whereas patients in the MHTT and LT cohorts were similar in their comorbidity profile. Patients in the LTT and MHTT cohorts had similar all-cause HRU; patients in the ST cohort had significantly higher all-cause HRU.

Limited treatment options are available for young children with an inadequate response to topical therapies. Prior to the approval of dupilumab for use in children, systemic therapies were often used off-label and contrary to treatment guidelines. In this study, a substantial portion (almost half of the study population) used SCS despite well-documented safety concerns.^11^, ^12^, ^13^ Alternative treatment options, including biologics, should be considered for children with difficult-to-treat AD.

More than half of the children with AD in the IQVIA PharMetrics Plus Database who were eligible for inclusion were excluded because no treatment for AD was reported. This may be due to several factors, including use of over-the-counter therapies, receipt of treatment samples from their physician, or fear of the potential adverse events associated with short- and long-term use of TCS/SCS.^11^, ^12^, ^13^ Furthermore, using different potencies of TCS may not be an effective way of differentiating moderate from mild cases in retrospective observational studies, as TCS use may be affected by the fear of using steroids. This possibility is evident from the findings of this study, in that HRU and costs were similar in patients receiving only low-potency TCS versus those receiving medium-to-high-potency TCS.

We found that the prevalence of atopic comorbidities was high across all cohorts, with the highest prevalence seen in the ST cohort; this was associated with higher overall/AD-related healthcare costs, generally consistent with previous studies.^14^^,^^15^ This is also consistent with findings that the AD onset is associated with the development of other type 2 comorbidities,^16^ with more severe disease associated with high likelihood^14^; AD-related healthcare costs were a small fraction of total healthcare costs, which could be indicative of increased healthcare costs related to the high prevalence of atopic comorbidities. Dupilumab has been approved for the treatment of a few other type 2 immune conditions^5^ and was associated with potentially preventing the development of other type 2 diseases.^17^

Numerous studies have shown the economic impact of AD among adults and adolescents in different regions of the world,^18^, ^19^, ^20^, ^21^, ^22^ in line with our findings in pediatric population in the United States.

### Strengths

This study had several strengths. It quantified disease burden in terms of HRU and healthcare costs in pediatric patients aged ≥6 months to <6 years with AD in the United States. To date, no studies have published a similar analysis for this population across disease severity. The large claims database provides access to a substantial number of patients with AD in the United States, with a probable wide distribution of comorbidities and treatment patterns; furthermore, the data used in this analysis provide an adequate representation of all areas across the United States and different types of health insurance. Adjusted analyses accounted for potential confounders in patients’ characteristics across cohorts, such as comorbidities.

### Limitations

The study also had several limitations. A selection bias may have been introduced by requiring 1.5 years of continuous enrollment, and patients fulfilling this criterion may be healthier than those who were excluded. Furthermore, patients who used over-the-counter treatments were not included in the analysis, as these therapies are not captured by claims data; there may have been some misclassification if treatments were used for conditions other than AD (eg, asthma). As we did not have data on treatment duration, we cannot speculate on treatment adherence. Additionally, patients’ out-of-pocket costs were not assessed, potentially introducing selection bias. Certain socioeconomic groups may be unrepresented if such groups have out-of-pocket costs that may be too high to drive the families to seek care, therefore limiting generalizability of the results. Since the observation period was before the availability of biologic treatments in this age group, these results do not reflect the situation after biologic treatment approval for use in children aged <6 years. The introduction of novel STs would likely increase the pharmacy costs while providing substantial benefits over existing STs. Future research should focus on the risk-benefit assessment of new STs, taking into consideration the different modes of action, and allowing physicians and healthcare policy decision makers to recommend the best treatment for their patients.

Finally, the study included only patients across the United States, limiting our conclusions to a specific population. Further studies should evaluate the generalizability of these findings in representative cohorts across the world.

## Conclusion

All pediatric patients with AD had a large number of atopic comorbidities, such as FA, asthma, and AR; the burden was highest in patients receiving ST. The high disease burden of AD and other atopic comorbidities was associated with high HRU and costs, particularly in the ST patient cohort, with medical costs being the main driver. A substantial proportion of patients were treated with SCS, with known adverse effects, at least partially explained due to high prevalence of comorbid asthma and lack of safe advanced ST options.

## Conflicts of interest

Dr Silverberg has been a consultant and/or received grants/honoraria from AbbVie, AnaptysBio, Asana BioSciences, Eli Lilly, Galderma, Glenmark, GSK, Kiniksa Pharmaceuticals, LEO Pharma, MedImmune, Menlo Therapeutics, Pfizer, PuriCore, Regeneron Pharmaceuticals Inc., and Sanofi. Drs Martins, Du, and Yang are employees of Analysis Group, a company that received research funds from Sanofi/Regeneron Pharmaceuticals Inc during the conduct of the study. Author Noonan is an employee of Sanofi and may hold stock and/or stock options in the company. Drs Wang and Korotzer are employees and shareholders of Regeneron Pharmaceuticals Inc.

## References

[1] Silverberg J.I., Simpson E.L. (2013). Association between severe eczema in children and multiple comorbid conditions and increased healthcare utilization. Pediatr Allergy Immunol.

[2] Al-Naqeeb J., Danner S., Fagnan L.J. (2019). The burden of childhood atopic dermatitis in the primary care setting: a report from the Meta-LARC Consortium. J Am Board Fam Med.

[3] Silverberg J.I., Barbarot S., Gadkari A. (2021). Atopic dermatitis in the pediatric population: a cross-sectional, international, epidemiologic study. Ann Allergy Asthma Immunol.

[4] Chu D.K., Schneider L., Asiniwasis R.N., AAAAI/ACAAI JTF Atopic Dermatitis Guideline Panel (2024). Atopic dermatitis (eczema) guidelines: 2023 American Academy of Allergy, Asthma and Immunology/American College of Allergy, Asthma and Immunology Joint Task Force on Practice Parameters GRADE- and Institute of Medicine-based recommendations. Ann Allergy Asthma Immunol.

[5] Dupilumab PI (EU): European Medicines Agency. DUPIXENT® (dupilumab). Summary of product characteristics. https://dailymed.nlm.nih.gov/dailymed/drugInfo.cfm?setid=595f437d-2729-40bb-9c62-c8ece1f82780.

[6] Paller A.S., Siegfried E.C., Simpson E.L. (2021). A phase 2, open-label study of single-dose dupilumab in children aged 6 months to <6 years with severe uncontrolled atopic dermatitis: pharmacokinetics, safety and efficacy. J Eur Acad Dermatol Venereol.

[7] Paller A.S., Siegfried E.C., Simpson E.L. (2024). Dupilumab safety and efficacy up to 1 year in children aged 6 months to 5 years with atopic dermatitis: results from a phase 3 open-label extension study. Am J Clin Dermatol.

[8] Wollenberg A., Ikeda M., Chu C.Y. (2024). Longer-term safety and efficacy of baricitinib for atopic dermatitis in pediatric patients 2 to <18 years old: a randomized clinical trial of extended treatment to 3.6 years. J Dermatolog Treat.

[9] IQVIA PharMetrics® plus database. https://test-www.iqvia.com/.

[10] Paller A.S., Siegfried E.C., Vekeman F. (2020). Treatment patterns of pediatric patients with atopic dermatitis: a claims data analysis. J Am Acad Dermatol.

[11] Ricci G., Dondi A., Patrizi A., Masi M. (2009). Systemic therapy of atopic dermatitis in children. Drugs.

[12] Sidbury R., Davis D.M., Cohen D.E., American Academy of Dermatology (2014). Guidelines of care for the management of atopic dermatitis: section 3. Management and treatment with phototherapy and systemic agents. J Am Acad Dermatol.

[13] Slater N.A., Morrell D.S. (2015). Systemic therapy of childhood atopic dermatitis. Clin Dermatol.

[14] Paller A.S., Spergel J.M., Mina-Osorio P., Irvine A.D. (2019). The atopic march and atopic multimorbidity: many trajectories, many pathways. J Allergy Clin Immunol.

[15] Ortsäter G., De Geer A., Rieem Dun A. (2022). Societal economic burden and determinants of costs for atopic dermatitis. JEADV Clin Pract.

[16] Gabryszewski S.J., Dudley J., Shu D. (2023). Patterns in the development of pediatric allergy. Pediatrics.

[17] Bauer A.H., Yadav S.R., Chen C.B. (2024). Use of dupilumab in pediatric patients: a review. Curr Pediatr Rep.

[18] Ricci G., Bendandi B., Pagliara L., Patrizi A., Masi M. (2006). Atopic dermatitis in Italian children: evaluation of its economic impact. J Pediatr Health Care.

[19] Ariëns L.F.M., van Nimwegen K.J.M., Shams M. (2019). Economic burden of adult patients with moderate to severe atopic dermatitis indicated for systemic treatment. Acta Derm Venereol.

[20] Elezbawy B., Fasseeh A.N., Fouly E. (2022). The humanistic and economic burden of atopic dermatitis among adults and adolescents in Saudi Arabia. J Med Econ.

[21] Elezbawy B., Fasseeh A.N., Fouly E. (2023). Humanistic and economic burden of atopic dermatitis for adults and adolescents in the Middle East and Africa region. Dermatol Ther (Heidelb).

[22] Achten R., Van der Rijst L., Piena M. (2023). Economic and humanistic burden in paediatric patients with atopic dermatitis. Acta Derm Venereol.

